# Novel treatment of bronchopleural fistula in a lung transplant recipient with endobronchial sealant and endobronchial valve placement via robotic-assisted bronchoscopy

**DOI:** 10.1093/jscr/rjad383

**Published:** 2023-07-08

**Authors:** Ishaq J Wadiwala, Sebastian Fernandez-Bussy, Pankaj Garg, Mostafa Ali, Neil G Feinglass, Mathew Thomas, Si M Pham

**Affiliations:** Cardiothoracic Surgery Research Unit, Mayo Clinic, Jacksonville, FL, USA; Division of Pulmonary and Critical Care Medicine, Mayo Clinic, Jacksonville, FL, USA; Department of Cardiothoracic Surgery, Mayo Clinic, Jacksonville, FL, USA; Cardiothoracic Surgery Research Unit, Mayo Clinic, Jacksonville, FL, USA; Department of Anesthesiology, Mayo Clinic, Jacksonville, FL, USA; Department of Cardiothoracic Surgery, Mayo Clinic, Jacksonville, FL, USA; Department of Cardiothoracic Surgery, Mayo Clinic, Jacksonville, FL, USA

## Abstract

Bronchopleural fistulas (BPFs) are a dreaded complication following pulmonary surgery. Endobronchial valves (EVs), with endobronchial sealant (ES), instilled with robotic bronchoscopy (RB), allow occlusion of BPF, avoiding surgery. The patient was a 71-year-old woman with a history of chronic obstructive pulmonary disease and bronchiectasis who underwent bilateral lung transplantation and wedge resection of the right middle lobe and left lingula. A BPF was discovered on postoperative day (POD) 21. Conservative measures with chest tubes failed, and robotic-assisted bronchoscopy aided in reaching the bronchial segment and instilling ES, and EV was deployed with the conventional bronchoscope. The pneumothorax was cleared 12 days later, and on POD 56, she was discharged. The RB procedure was successful, with no pneumothorax or BPF symptoms after a median follow-up of POD 284. Robotic endobronchial closure of BPF with EV and ES is an effective treatment option avoiding invasive surgeries.

## INTRODUCTION

Bronchopleural fistula (BPF) after lung transplant is infrequent [1]. Currently, endobronchial closure of BPF using glues, coils and valves is the treatment of choice; however, it is limited by the inability of conventional bronchoscopes to access and stabilize in smaller airways [2]. Robotic-assisted bronchoscopy (RAB) permits navigation through smaller airways under direct vision and electromagnetic guidance (MonarchTM platform by Auris Health Inc) or Shape Sensing Technology (IonTM Endoluminal System by Intuitive Surgical), helping localize target airways and providing stability during delivery of occluding material [3]. We reported a lung transplant recipient with BPF that was successfully treated with placement of sealant by RAB and the endobronchial valve was placed via a traditional bronchoscope as an adjunct therapy.

## CASE REPORT

**Figure 1:**
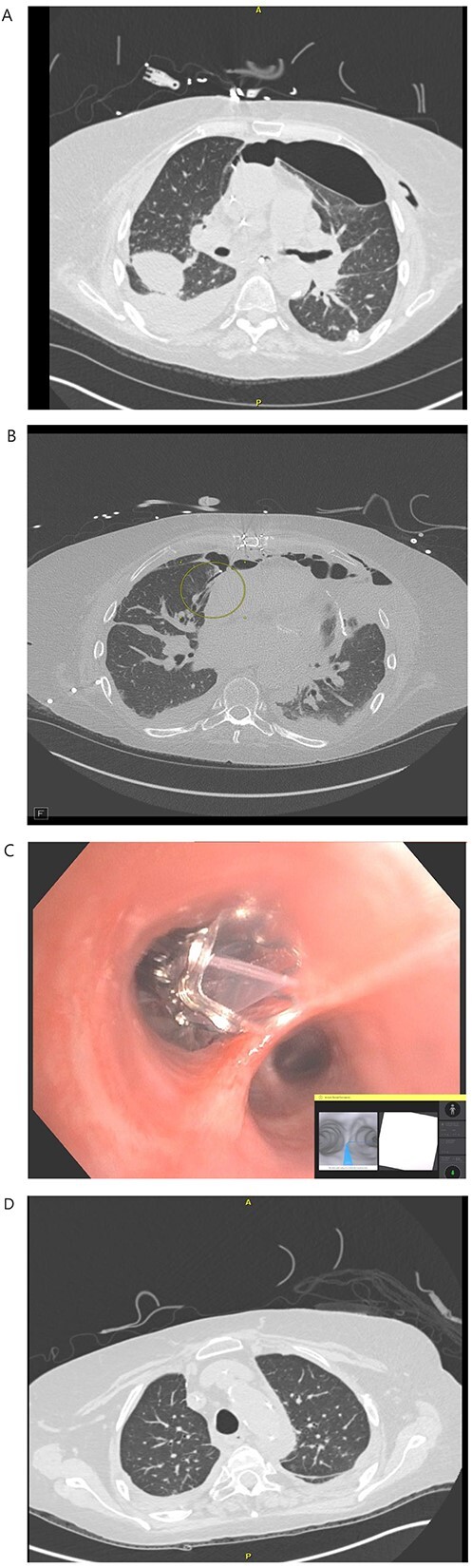
(**A**) POD 21 BL pneumothorax and R pleural effusion. (**B**) POD 31 BPF with medial segment RML with BL pneumothorax and R hydropneumothorax. (**C**) POD 35 placement of endobronchial valve in medial segment of RML. (**D**): POD 41 resolution of BPF, pleural effusion and pneumothorax.

A 71-year-old female with end-stage lung failure underwent a bilateral lung transplant with wedge resection of the right middle lobe and left lingular of donor lungs using Endo-GIA staplers due to oversized lungs. On postoperative day (POD) 21, a BPF was noticed from the right middle lobe at the staple line (Fig. 1B) with persistent bilateral pneumothorax and a loculated right-sided pleural effusion (Fig. 1A) on chest computed tomography (CT) scan that failed conservative treatment with multiple chest tubes. The patient underwent RAB-assisted BPF for closure of BPF on POD 35 using IonTM Endoluminal System. RAB was used to locate and reach the medial lobe bronchial segment of the right middle lobe (Fig. 1C), and a combination of Histoacryl and lipiodol were instilled. The RAB system was then withdrawn, and traditional bronchoscopy was used to deploy the Zephyr® endobronchial valve (Pulmonx, Redwood City, CA) in the medial segment bronchus. An uneventful recovery after the procedure with an improved CT chest (Fig. 1D) was noticed and eventually discharged on POD 57. The EBV was removed on POD 133. At the last follow-up 1-year post transplant, the patient can carry out her daily activities at home without oxygen support.

## DISCUSSION

RAB has the potential to increase accessibility to peripheral lung lesions. Due to the smaller size of bronchoscopes, electromagnetic or shape sensing technology and the stable instrumentation platform provided by robots, RAB allows the direct visualization of up to the ninth airway segment lesions, including BPF [3]. We chose to use ‘The IonTM Endoluminal System’ because the shape-sensing technology of the video probe allowed live visualization and safe advancing the catheter safely to the proximity of the feeding bronchus.

As the late BPF is challenging to treat, we preferred Histoacryl and lipiodol instead of Progel (BD, Crystal Lake, NJ) to provide more permanent closure. The EBV (which was placed using a conventional bronchoscope) allows air expiration from a treated bronchus but prevents re-inspiration, thus limiting the reopening of the BPF and facilitating closure [4, 5].

Bronchoscopic intervention for small BPFs using the RAB system is a valuable therapeutic alternative that improves the precision of localization and intervention in the areas difficult to access surgically or with conventional bronchoscopy.

## Data Availability

Data available on request from the authors.
